# Synergistic combination of two antimicrobial agents closing each other’s mutant selection windows to prevent antimicrobial resistance

**DOI:** 10.1038/s41598-018-25714-z

**Published:** 2018-05-08

**Authors:** Xuejie Xu, Li Xu, Ganjun Yuan, Yimin Wang, Yunqiu Qu, Meijing Zhou

**Affiliations:** 1 College of Bioscience and Bioengineering, Jiangxi Agricultural University, Nanchang, 330045 China; 2 Affiliated Hospital, Jiangxi Agricultural University, Nanchang, 330045 China

## Abstract

Antimicrobial resistance seriously threatened human health. Combination therapy is generally an effective strategy to fight resistance, while some data on its effects are conflicting. To explore the reasons, the fractional inhibitory concentration indexes (FICIs) of three designed combinations against methicillin-resistant *Staphylococcus aureus* (MRSA) were determined using checkerboard method, and their minimal concentrations inhibiting colony formation by 99% (MIC_99%_s) and mutant prevention concentrations (MPCs) alone or in combinations including different proportions were first determined using agar plates. The results indicated that different proportions of a combination had presented different MPCs and mutant selection window (MSWs), and also showed that the smaller the FICIs of two agents in combinations were, the more probable their MSWs were to close each other. As two agents of a combination had different pharmacokinetic characters, the ratios of two agents in blood and infectious sites were likely different even though a specific proportion was administrated, which would lead to different effects preventing resistance. Thereby, these experimental results theoretically indicated that synergistic combination closing each other’s MSWs had a great potency to prevent resistance according to the hypotheses of MSW and MPC, and deduced that *in vivo* synergistic validity of a combination was likely a key to prevent resistance. Moreover, a synergistic combination of roxithromycin/doxycycline with the FICIs of 0.26–0.50 and 0.28–0.38 respectively against MRSA 01 and 02 was obtained, and the MSWs of these two agents could be simultaneously closed each other in a certain range of proportions, but for others. Meanwhile, its effect preventing resistance needs to be further verified.

## Introduction

As multi-drug resistance organisms including “ESKAPE” pathogens had been spreading widely and seriously threatened human health^1–3^, many strategies involving development of new antimicrobial agents^3^, revival of old antibiotics^4^, combination therapy and optimal use of clinic antimicrobial agents had been putting forward to fight or delay resistance^5^. Considering that many microorganisms would be found to be resistant to a new antimicrobial agent soon after used in clinic practices, our group had a great interest to combination therapy when we tried our best to discover new antimicrobial agents^6–8^. Many combinations had been studying for synergy *in vitro* and *in vivo*^9^, while some data on the therapeutic effects of them to prevent resistance are conflicting^10–13^. Even more, some combinations may result in high mutational frequencies, such as levofloxacin in combination with lower dose of colistin^14^.

According to the hypotheses of mutant selection window (MSW) and mutant prevention concentration (MPC) put forward by Zhao and Drlica^15,16^, maintaining drug concentrations above its MPC throughout therapy can severely restrict the acquisition of drug resistance and achieve its therapeutic effect, while this will increase the risk of adverse and toxic effects. Simultaneously, the drug concentration will unavoidably fall into its MSW (The range from minimal concentration inhibiting colony formation by 99% (MIC_99%_) to MPC). Inspired by the analyses of combination therapies reported^12–14,17,18^, we empirically deduced that synergistic validity was likely a key to prevent antimicrobial resistance and balance these factors during combination therapy. Theoretically, the more remarkable the synergistic effect of two antimicrobial agents in a combination was, the more probable their MSWs were to close each other. Thereby, we may discover synergistic combination closing each other’s MSWs to avoid their drug concentrations falling into its MSW as possible as we can, and obtain content therapeutic effect with lower dose.

Closing MSW mean that mutant selection index (SI, the ratio of MPC to MIC_99%_) were less than or equal to one^16^. Are there some correlations between SI and fractional inhibitory concentration index (FICI) used to evaluate the synergistic effects of antimicrobial agents in checkerboard assay? To discover synergistic combinations as quickly as possible and explore the feasibility that synergistic combination closing each other’s MSWs to prevent antimicrobial resistance, three combinations including seven proportions of each combination were selected for clarifying the correlations between SI and FICI.

## Results

### Minimal inhibitory concentration (MIC) and checkerboard assay

Five antimicrobial agents roxithromycin (RM), doxycycline (DC), vancomycin (VM), fosfomycin (FF) and ofloxacin (OX) were selected for three combinations RM/DC, VM/OX and VM/FF including seven different proportions of each combination, and clinic isolates methicillin-resistant *Staphylococcus aureus* (MRSA) 01, 02 and 03 were used as pathogen bacteria for the MIC test and checkerboard assay. The MICs of five antimicrobial agents and FICIs of three combinations against three clinical MRSA isolates were showed in Table 1. The results indicated that MRSA 01 and 02 were susceptible to RM while MRSA 03 was resistant to RM, and that all three MRSA isolates were resistant to FF. Checkerboard assays showed that combination RM/DC presented remarkably synergistic activities against MRSA 01 and 02 with the FICIs of 0.26–0.50 and 0.28–0.38, but against MRSA 03 with that of 0.53–0.75. Other combinations with the FICIs of 0.75 to 1.50 showed no synergistic activity.

**Table 1:** Minimal inhibitory concentrations (MICs) of five agents and fractional inhibitory concentration Indices (FICIs) of three combinations against three clinical MRSA isolates^a^.

MRSA isolates	MIC (μg/mL)					FICI		
	RM^b^	DC	VM	OX	FF	RM/DC	VM/OX	VM/FF
01	0.13	0.25	1.00	1.00	64	0.26–0.50	0.75–1.25	1.00–1.25
02	0.13	0.13	2.00	2.00	32	0.28–0.38	1.00–1.50	1.25–1.50
03	32	0.13	0.50	2.00	32	0.53–0.75	1.25–1.50	1.00–1.25

^a^The data of FICI were published as an abstract of oral presentation at ISAAR&ICIC 2017^42^.

^b^RM: roxithromycin, DC: doxycycline, VM: vancomycin, FF: fosfomycin, OX: ofloxacin.

### MIC_99%_ alone and MPC alone or in combinations

MIC_99%_s alone and MPCs alone or in combinations of above antimicrobial agents were showed in Table 2. As previous papers reported^16,19–21^, no obvious correlations between MIC (or MIC_99%_) and MPC alone were also observed from Tables 1 and 2. Thereby, we couldn’t use MIC (or MIC_99%_) to predict MPC. All the MPCs of antimicrobial agents in combinations decreased in different degrees regardless of synergy or indifference presented in checkerboard assays. However, the larger MPC of two agents in a combination reduced more remarkably than the smaller one. Among them, the MSWs of some proportions of combination RM/DC against MRSA 01 and 02 could close each other, but those against MRSA 03 being resistant to RM. These indicated that synergistic combinations of antimicrobial agents being susceptible to pathogenic bacteria had a great potency to prevent resistance according to the hypotheses of MSW and MPC.

**Table 2:** MIC_99%_s and MPCs of antimicrobial agents alone and their MPCs in combinations^a^.

MRSA isloates	Combinations^a^	MIC_99%_s Alone	MPCs Alone	MPCs in combinations^c^						
				8:1	4:1	2:1	1:1	1:2	1:4	1:8
01	RM/DC	0.04/0.17	0.21/2.56	0.09/0.01	0.10/0.02	0.07/0.03	0.08/0.08	0.03/0.05	0.04/0.15	0.01/0.12
	VM/OX	0.32/0.92	15.4/25.6	4.56/0.57	9.01/2.25	4.44/2.22	2.72/2.72	1.79/3.58	1.44/5.76	0.73/5.84
	VM/FF	0.32/6.92	15.4/1000	4.56/0.57	3.57/0.89	4.44/2.22	3.54/3.54	8.70/17.4	4.36/17.4	4.38/35.0
02	RM/DC	0.07/0.10	0.16/2.00	0.08/0.01	0.07/0.02	0.05/0.03	0.05/0.05	0.04/0.09	0.03/0.13	0.02/0.16
	VM/OX	1.79/1.12	16.0/19.2	8.88/1.11	7.04/1.76	5.97/2.99	2.88/2.88	2.83/5.67	1.40/5.60	1.67/13.4
	VM/FF	1.79/8.01	16.0/1000	9.36/1.17	9.22/2.30	4.50/2.25	4.61/4.61	3.77/7.55	5.76/23.0	2.25/18.0
03	RM/DC	0.28/0.04	256/0.39	2.64/0.33	1.34/0.34	0.86/0.43	0.41/0.41	0.20/0.41	0.11/0.42	0.07/0.56
	VM/OX	0.21/1.25	15.0/8.00	1.76/0.22	4.40/1.10	2.04/1.02	2.00/2.00	1.49/2.99	0.77/3.07	0.53/4.24
	VM/FF	0.21/10.3	15.0/256	4.48/0.56	7.04/1.76	4.34/2.17	5.44/5.44	4.25/8.50	4.25/17.0	4.28/32.2

^a^Most data were published as an abstract of oral presentation at ISAAR&ICIC 2017^42^.

^b^RM: roxithromycin, DC: doxycycline, VM: vancomycin, FF: fosfomycin, OX: ofloxacin.

^c^MPCs of two antimicrobial agents in three combinations with seven different proportions.

### SIs of two agents in different proportions of a combination presented different FICIs against MRSA

**Figure 1:**
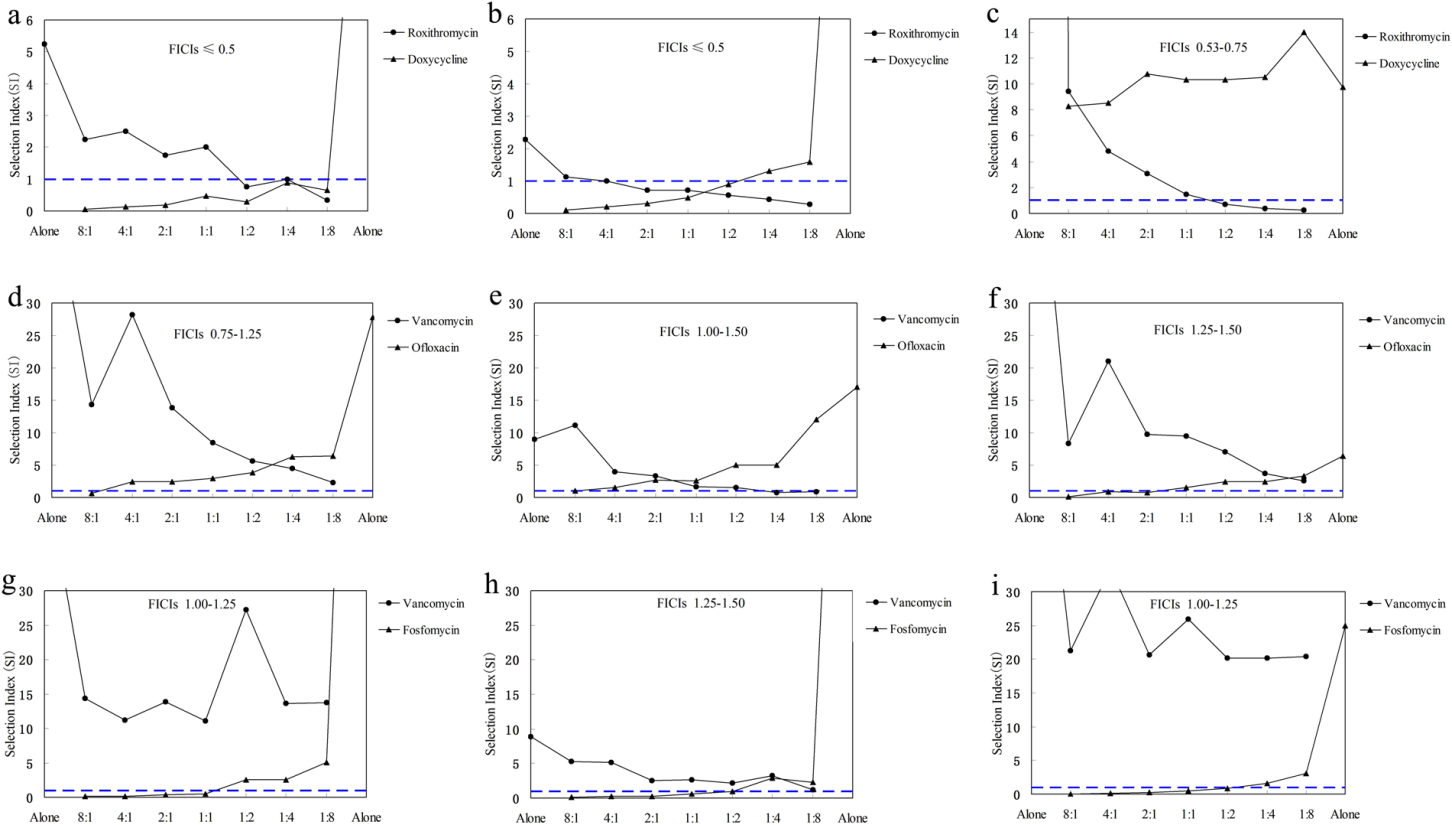
Mutant selection indexes (SIs) of antimicrobial agents in three combinations with different fractional inhibitory concentration indexes (FICIs) against three clinic MRSA isolates. (**a**–**c**), combination roxithromycin/doxycycline against clinical isolates MRSA 01, 02 and 03; (**d**–**f**), combination vancomycin/ofloxacin against clinical isolates MRSA 01, 02 and 03; (**g**–**i**), combination vancomycin/fosfomycin against clinical isolates MRSA 01, 02 and 03. That the FICIs of combinations were less than or equal to 0.50 meant that two agents in combinations presented synergistic activities against a specific MRSA isolate. That the SIs of two agents in a combination including seven proportions were simultaneously less than or equal to one represented their MSWs were closed each other. (**a**–**f**) were published as an abstract of poster presentation at ISAAR&ICIC 2017^43^.

Regardless of the effect of host defense^22^, maintaining drug concentrations above MPC throughout therapy can prevent resistance according to MSW and MPC hypotheses^16^. That meant that closing MSW could avoid the drug concentration falling into MSW, and that a combination in which SI of each agent was less than or equal to one would be efficacious to prevent antimicrobial resistance. To discover synergistic combinations as quickly as possible and easily predict the effect of preventing antimicrobial resistance, SIs of two agents in different proportions of each combination presented different FICIs against three MRSA isolates were graphed using data in Tables 1 and 2, and showed in Fig. 1 for presenting more clearly. Some information was easily observed as follows: (1) The larger MSW of two agents in a combination was prior to be closed; (2) The smaller the FICIs of two agents were, the more probable both SIs were less than or equal to one, and the wider the proportional ranges of two agents closing each other’s MSWs were (Fig. 1a,b); (3) Only the minimum FICI was less than or equal to 0.50, some proportions of two agents closing each other’s MSWs could be sought out from a combination; (4) Different proportions of two agents in a combination presented different SIs; (5) Synergistic combinations closing each other’s MSWs had a great potency to prevent or delay resistance according to MSW and MPC hypotheses.

## Discussion

Combination antimicrobial therapy with two or more drugs is a standard one for infections with human immunodeficiency virus and *Mycobacterium tuberculosis*^9–11^. As the development of new antimicrobial agents has been late for resistance, combination therapy had been also considering as an effective strategy to prevent antimicrobial resistance^23,24^, while some reports on their therapeutic effects to prevent resistance are conflicting^9^. Theoretically, the more remarkable the synergistic activity of two agents in a combination was, the more probable their MSWs were to close each other. Thus, discovering remarkably synergistic combinations closing each other’s MSWs were our goals.

**Figure 2:**
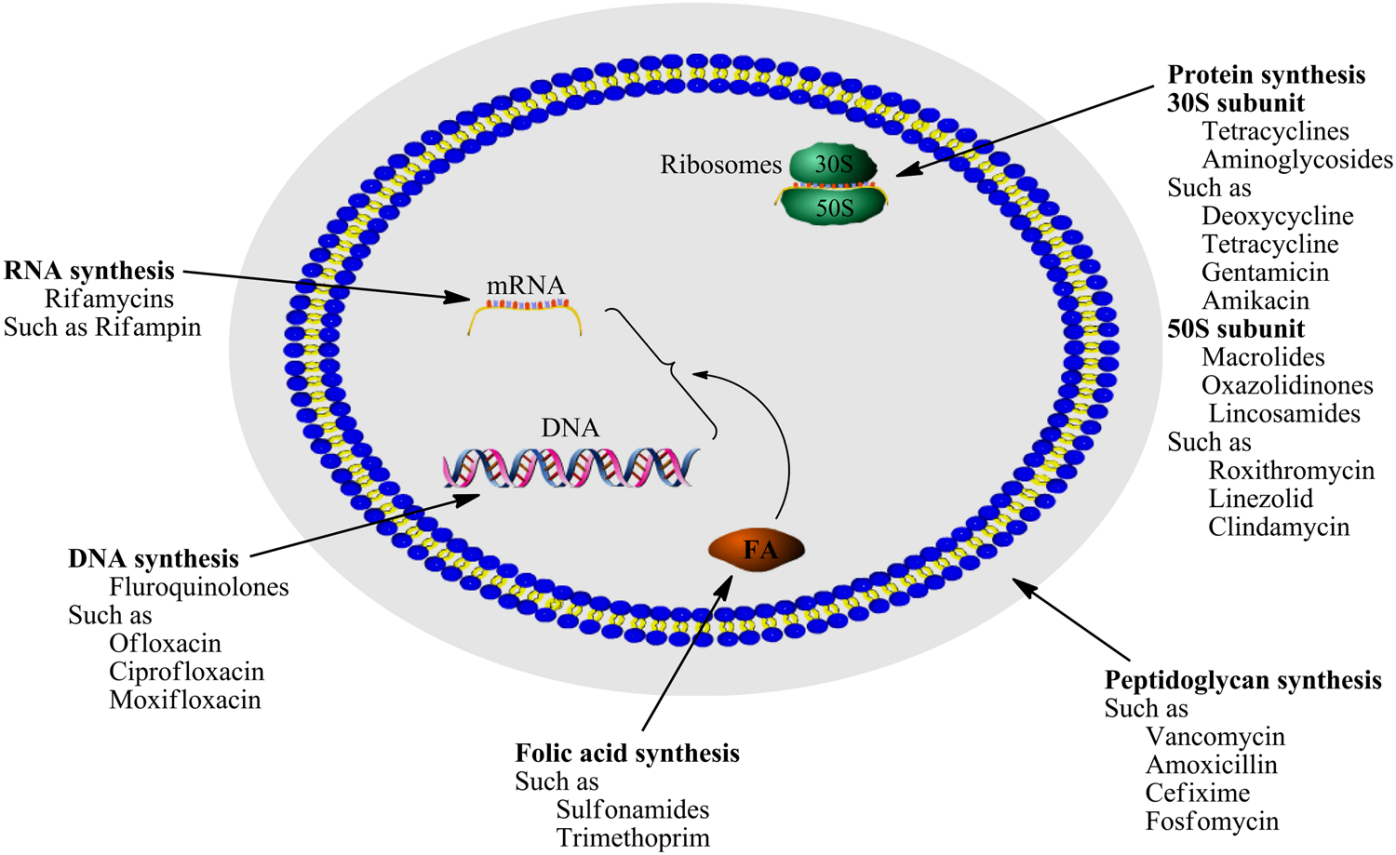
Main macromolecular targets involving antimicrobial mechanisms. This figure was drawn successively using Pathway Builder Tool 2.0, ChemBioOffice 2010 and Adobe Photoshop CS3.

The main targets of antibacterial agents include DNA, RNA, protein and peptidoglycan (Fig. 2). Analysis of various combinations reported^12–14,17,18^, we empirically put forward a tentative idea that antimicrobial agents targeting identical macromolecular biosynthesis with different sites likely presented remarkable synergistic activities. Thereby, RM, DC, VM, OX and FF were selected for three combinations RM/DC, VM/OX and VM/FF including seven proportions of each combination. In accord with our tentative idea, checkerboard assays (Table 1) indicated the combination of RM and DC, respectively targeting ribosomal protein 50*S* and 30*S* subunits, showed remarkably synergistic activity against MRSA 01 and 02 while that against MRSA 03 presented indifferent activities. The latter was likely due to MRSA 03 being resistant to roxithromycin. These above indicated that antimicrobial effects of two agents in a combination related to their susceptibilities to pathogenic bacteria, which had been further proving by previous researches^14^. This might be partly responsible for the conflicting results of combination therapy, and reminded us that it was better to administer in combinations before pathogenic bacteria were resistant to an antimicrobial agents.

To prevent resistance, many combinations were reported^12–14,17,18,25^, while different proportions of two agents in a combination were rarely determined. Twenty-one proportions of three combinations RM/DC, VM/OX and VM/FF were designed based on their checkerboard assays, and the results (Table 2) showed that different proportions of two antimicrobial agents in a combination would present different MPCs. The MPCs of two agents in a combination decreased in different degrees regardless of synergy or indifference in their checkerboard assays, and the larger MPC alone of two agents in a combination reduced more remarkably than the smaller one (Fig. 1). Simultaneously, the MPC values of anyone in an antimicrobial combination could be decreased to less than or equal to MIC_99%_ by increasing the proportion of another agent whether it’s synergy or not. This was also confirmed by previous researches on many combinations such as tigecycline and colistin or sulbactam^13^, levofloxacin and colistin^14^, and vancomycin and fosfomycin^17^. For example, the MSWs of tigecycline was closed by increasing the concentrations of colistin or sulbactam in combinations, while the MSWs of colistin or sulbatam were not simultaneously closed probably except for tigecycline in combination with sulbactam against *Acinetobacter baumannii* isolates Ab2 and Ab65^13^. Another, some MSWs of levofloxacin against three *Pseudomonas aeruginosa* isolates were closed by increasing the concentrations of meropenem, colistin, piperacillin-tazobactam, ceftazidime or tobramycin, while all MSWs of these antimicrobial agents combined with levofloxacin were not simultaneously closed^14^. As the FICIs of these combinations were unavailable^13,14^, we couldn’t judge whether these combinations are synergistic or not, while we had enough confidence to infer that the MSWs of two agents in a non-synergistic combination could be not closed each other (Fig. 1). This was also proved by vancomycin in combination with fosfomycin, levofloxacin or rifampicin^17^. Could synergistic combination closing each other’s MSWs completely prevent resistance? Even though combination RM/DC presented remarkably synergistic activities against MRSA 01 and 02, only proportions (1:2 to 1:8) against MRSA 01 and proportions (4:1 to 1:2) against MRSA 02 could close each other’s MSWs (Fig. 1a,b). That meant only applying proportion (1:2) could simultaneously prevent MRSA 01 and 02 being resistant to roxithromycin and doxycycline according to the hypotheses of MSW and MPC. This indicated that an unreasonable combination was likely to lead pathogenic bacteria to be resistant to one susceptible agent when it enhanced the susceptibility of another agent. Furthermore, human body is a complex system, and the antimicrobial and resistance-prevented effects were influenced by various factors including human body (defense system and microorganism communities), antimicrobial agents (drug concentrations in blood and infection site, pharmacokinetics, toxic side effect to human body including immune suppression, and selective antimicrobial or bactericidal effect to destruct the balance of microorganism communities which were favorable to prevent infection and resistance) and pathogenic bacteria (biofilm, persisters, and resistant mutants). Although synergistic combination RM/DC theoretically had a great potency to prevent resistance of two isolates MRSA 01 and 02 in a wide proportionality range, the actual effect preventing resistance of combination RM/DC with different proportions needs to be further verified using pharmacokinetic simulations on *in vitro* model and those of bacterial biofilm^26,27^.

As two antimicrobial agents in a combination usually presented different pharmacokinetics parameters *in vivo*, their proportions in blood and infectious tissue would accordingly change, and thus lead to their different MPC and SI values. This must fluctuate or even invert the practical effects preventing resistance, and was likely another important reason for the conflicting results during combination therapy^9,18^. Thereby, we might select two agents with similar pharmacokinetics parameters as possible as we could for synergistic combination to prevent resistance^16^. Furthermore, the more significant the synergistic effect of two agents in a combination was, the wider the proportional range of two agents closing each other’s MSW was. Thereby, remarkably synergistic combinations would be more favorable to prevent resistance.

In accord with what we had reported^8^, that the correlation between the ratio of two agents in a combination and the sum of their SIs (the ratio of MPC in combination to MIC_99%_ alone) could be expressed as a binary regression equation. Using this regression equation, we can predict the SIs and MPCs of one agent from those of another in different proportions, and finally deduce the perfect ratio of two agents in a combination and their potency to prevent resistance. The roughly positive correlations (observed from Fig. 1) between SI and FICI indicated that the smaller the FICI value of two agents was, the more probable the combination was to close each other’s MSW (SI was less than or equal to one). This showed synergistic combinations had a great potency and were more helpful to prevent resistance. Simultaneously, FICI used for evaluating the synergistic effects of two antimicrobial agents could be easily determined using checkerboard method on 96-well plates, while the determination of SI used for evaluating the potential effect of antimicrobial agents to prevent resistance were more complicated and would spend a lot of time. Thereby, we can select two agents with the FICI value as small as possible for combinations to prevent resistance. Perfectly, the maximum FICI value was less than 0.50, even 0.38.

As the MSWs of one antimicrobial agent can be narrowed to some extent by combining with another whether it is synergistic or not, many combinations had enough potential to prevent resistance^14,17,25,28^, and which mainly depended on their dose administrated and the change of their MSWs in a combination. Even, these could partly interpret why the susceptibility of one antimicrobial agent might be enhanced by another in an antagonistic combination^29^. However, synergistic combination of two agents closing each other’s MSW (namely SI ≤1) can prevent resistance regardless of their therapy dose according to the hypothesis of MSW, and just the change of their MSWs as different proportions presented different MPCs of two agents in a combination need to be considered. Simultaneously, Fig. 1a–c, and Tables 1 and 2 roughly showed that the remarkable the synergistic effect is, the narrower their MSWs are, and the wider the ratio range of two agents in a synergistic combination closing each other’s MSW. Thereby, the indifferent combination can prevent resistance by applying the same doses as they administrated alone or by increasing their doses^26^, while antimicrobial combination with FICIs as small as possible had greater and superior potency to effectively prevent resistance, which was also proved by previous paper^30^. Another, to reveal changes in susceptibility after treatment, the MIC of an antimicrobial agent against exposed bacterial colony was determined for accessing the effectiveness to prevent resistance, while no paper involved whether the MPC of that changes.

Although it was difficult to conclusively demonstrate the absolute advantages of combination therapy compared to single antimicrobial administration for treating bacterial infections, and even some clinical results were conflicting^9,16,18^, we should have enough confidence to believe that combination therapy had affirmable potency to prevent or delay antimicrobial resistance. Above experimental data and analyses showed that antimicrobial agents targeting identical macromolecular biosynthesis pathway with different mechanisms had a great potency to discover synergistic combinations. A synergistic combination of two agents with similar pharmacokinetics would be favorable to prevent resistance, especially when pathogenic bacteria were susceptible to them. Simultaneously, the maximum FICI of two agents in a synergistic combination was perfectly less than or equal to 0.38. Further, a new antimicrobial agent synergistically combining with one or more ones, as a regular combination, should be encouraged to be approved, and even as a hybrid antibiotic such as rifamycin-quinolone^31^.

Further, microorganisms in human body can promote or restrain each other’s growth to form balanced microorganism communities which is favorable for us to prevent infection and resistance^32^, and the MICs of an antimicrobial agent against different pathogens were different. Thereby, an antimicrobial agent can likely promote some pathogens growing, forming biofilm, complex biofilm and persisters, or even acquiring resistance for being tolerant to it when it kills or inhibits other pathogens^33–36^, and which are likely unfavorable for preventing resistance. Moreover, the toxic side effects of an antimicrobial agent are harmful to our defense system killing pathogens and eliminating toxin sometimes^32^. However, these deleterious influences of single antimicrobial agent can be largely decreased when its dosage reduced through synergistic combination therapy.

## Methods

### Isolates and medium

Three clinical MRSA isolates 01, 02 and 03 obtained from the Clinical Laboratory of the Second Affiliated Hospital, Sun Yat-sen University were stored at −20 °C in 20% glycerol^7^, and cultured on Mueller-Hinton Agar (MHA) slant at 35 °C. Pure colonies were cultured in 10 ml fresh Mueller-Hinton Broth (MHB) at 35 °C until the optical density (OD_600_) was 0.60 to obtain bacterial suspension. MHB used for antimicrobial susceptibility test and checkerboard assay, and MHA used for the measurements of MIC_99%_ and MPC were obtained from Qingdao Hope Bio-Technology Co., Ltd., Qingdao, China.

### Antimicrobial agents and chemicals

Roxithromycin (940 U/mg), doxycycline hydrochloride (88~94%) and vancomycin hydrochloride (98%) were purchased from BBI Life Sciences Corporation, Shanghai, China. Ofloxacin (97%) was purchased from Ark Pharm, Inc., Arlington Heights, USA. Fosfomycin disodium salt (99.5%) was purchased from Shanghai Macklin Biochemical Co., Ltd., Shanghai, China. All other solvents and agents with analytical grade were obtained from Sinopharm Chemical Reagent Co., Ltd., Shanghai, China.

### Antimicrobial susceptibility

The MICs of five antimicrobial agents against three clinical MRSA isolates were determined according to a standard procedure described by the Clinical and Laboratory Standards Institute (CLSI) in 2012^37^. The tests were performed using broth microdilution method on 96-well plates in triplicate, and the plates were incubated at 35 °C for 24 h. The MIC was defined as the lowest concentration of antimicrobial agent that completely inhibited bacterial growth in the microdilution wells as detected by the unaided eye when the bacterial growth in blank wells was sufficient.

### Checkerboard assay

Referring to the MICs of five agents, checkerboard assay was designed to determine their FICIs in combinations against three clinical MRSA isolates, and the tests were performed on 96-well plates according to previous methods^38,39^. Combination RM/DC was used an example for interpreting the test process. Briefly, RM and DC dilutions from 8 to 1/16 MIC in the horizontal or vertical direction were prepared in a separate 96-well plate by twofold dilution method. Next, 100 μl RM or DC dilutions with different concentrations were correspondingly added into the designed wells on another plate to obtain different proportions with RM or DC concentrations from 4 to 1/32 MIC. Another, columns 11 and 12 only contained MHB with 5 × 10^5^ cfu/ml MRSA isolate were used as blank controls. When the microbial growth in blank wells was good at 35 °C for 24 h, the MIC of each agent was defined as the lowest concentration of MRSA growth visibly inhibited. If necessary 3-(4,5-dimethylthiazol-2-yl)-2,5-diphenyltetrazolium bromide (MTT) stain method was used to clearly observe the results. The MICs of RM and DC alone were respectively observed in row A and in column 1, and the MICs of RM in combination with DC were obtained from wells B2 to H8.

The FICs were calculated as follows: FIC of RM = MIC of RM in combination/MIC of RM alone, and FIC of DC = MIC of DC in combination/MIC of DC alone. The FICI was defined as the FIC of RM plus the FIC of DC. The combining effect of RM in combination with DC against MRSA interpreted as follows: Synergy, FICI ≤ 0.5; Indifference, 0.5 < FICI > 4.0; and antagonism, FICI ≥ 4.0^40^.

### MIC_99%_ alone

According to the MICs alone of antimicrobial agents, their MIC_99%_s alone against three clinical MRSA isolates were determined in duplicate by agar plate methods described in previous papers^15,41^. In brief, MRSA suspension was plated on agar plates containing linear drug concentrations with 20% per sequential decrease from each MIC, and those plates containing no drug used for blank controls. The plates were incubated at 35 °C for 24 h, and then the colonies growing on different plates were numbered. Finally, inhibition percentage (*y*) was calculated, and was plotted against different antimicrobial concentrations (*x*) to obtain a regression equation for an antimicrobial agent. Accordingly, their MIC_99%_s were respectively calculated according to their individual equations.

### MPC alone or in combination

Using three clinical MRSA isolates, their MPCs alone or in combination were determined as described in previous reports^15,41^. Briefly, high-density cultures (approximately 5.0 × 10^10^ cfu/ml) were prepared from overnight MRSA cultures grown in MHB, and followed by a 10-fold dilution, incubation with shaking at 35 °C for 6 h, centrifugation, and adjustment with normal saline. According to the MIC of an antimicrobial agent, 100 μl culture containing about 5.0 × 10^9^ colony forming units were plated on a series of MHA plates containing an antimicrobial agent or combination with twofold dilution concentrations from 64 MIC, and the plates were incubated at 35 °C for 72 h. Then, the preliminary MPC was recorded as the lowest antimicrobial concentration that prevented bacterial growth. Further, the exact MPC was determined in duplicate by linear antimicrobial concentration with 20% per sequential decrease from preliminary MPC. Referring to the FIC values of five agents of three combinations RM/DC, VM/OX and VM/FF, the MPCs of twenty-one combinations were determined according to above methods and procedures.

### Data availability

The datasets generated during and/or analysed during the current study are available from the corresponding author on reasonable request.
